# Increasing and more consistent use of pre-biopsy MRI in prostate cancer diagnosis: insights from a population-based study in the Netherlands

**DOI:** 10.1186/s13244-026-02282-9

**Published:** 2026-05-28

**Authors:** C. C. Loeff, I. G. Schoots, P. J. van Leeuwen, D. E. Oprea-Lager, S. Al-Uwini, L. A. L. M. Kiemeney, I. M. van Oort, K. K. H. Aben, B. L. Heesterman

**Affiliations:** 1 Department of Research and Development, Netherlands Comprehensive Cancer Organization, Utrecht, The Netherlands; 2 Department of Medical Imaging, Radboud university medical center, Nijmegen, The Netherlands; 3 Department of Radiology and Nuclear Medicine, Erasmus University Medical Center, Rotterdam, The Netherlands; 4 Department of Radiology, Netherlands Cancer Institute-Antoni van Leeuwenhoek Hospital, Amsterdam, The Netherlands; 5 Department of Urology, Netherlands Cancer Institute-Antoni van Leeuwenhoek Hospital, Amsterdam, The Netherlands; 6 Department of Radiation Oncology, University Medical Center Groningen, Groningen, The Netherlands; 7 Science department IQ Health, Radboud university medical center, Nijmegen, The Netherlands; 8 Department of Urology, University Medical Center Groningen, Groningen, The Netherlands

**Keywords:** Hospital variation, Prostate biopsy, Prostate cancer, Prostate MRI, Magnetic resonance imaging

## Abstract

**Objective:**

Guidelines recommend MRI before prostate biopsy in men with suspected prostate cancer (PCa). However, real-world data on clinical use are scarce. This study aims to provide comprehensive insight into the nationwide use of pre-biopsy MRI across the Netherlands.

**Materials and methods:**

Men with biopsy-proven primary PCa diagnosed between 2019 and 2023, were identified through the Netherlands Cancer Registry (main cohort; *n* = 50,987). A historical cohort included similar cases from 2015 to 2016 (*n* = 5183). Four MRI-related periods were defined: historical (2015–2016), pre-implementation (2019), implementation (2020), and post-implementation (2021–2023). Mixed-effects logistic regression analyses assessed factors associated with pre-biopsy MRI use and heterogeneity across periods. Inter-hospital variation was quantified using case-mix (age, PSA, clinical disease stage) adjusted hospital-specific probabilities.

**Results:**

Pre-biopsy MRI use increased from 17 to 74% between 2015 and 2023. Across all periods (main cohort), men over 70 and those with a PSA > 50 µg/L or cT3-4 disease were significantly less likely to undergo pre-biopsy MRI than younger men, those with PSA < 10 µg/L, or cT1 disease, respectively. Heterogeneity in effect size across periods was observed for all factors except age. Inter-hospital variation was present in all MRI-related periods, although it significantly decreased over time. Estimated 75% midrange rates varied from 9.7–86% (pre-implementation) to 62–88% (post-implementation).

**Conclusions:**

Pre-biopsy MRI use in PCa diagnosis has markedly increased and has become more consistent across hospitals. Over time, its use became more targeted, focusing on patients without signs of advanced disease, in accordance with EAU guidelines recommendations. Continued efforts to standardize MRI use may improve equity and optimize patient outcomes.

**Critical relevance statement:**

This study evaluates the nationwide implementation of pre-biopsy MRI in PCa diagnosis, revealing increased and more targeted use, demonstrating consistency across hospitals, and providing insights to guide standardization and optimize patient outcomes.

**Key Points:**

- Comprehensive population-level data on the uptake of pre-biopsy MRI after implementing guideline recommendations are essential for clinical practice evaluations on a national level.
- In the Netherlands, these data show an increase in the uptake of pre-biopsy MRI over time, demonstrate consistent patterns across hospitals, and illustrate a shift towards use in patients without advanced disease, aligning with EAU guidelines recommendations.
- Ongoing efforts to implement and standardize pre-biopsy MRI use in routine clinical practice are critical to promote equity and optimize patient outcomes on a population-based level.

**Graphical Abstract:**

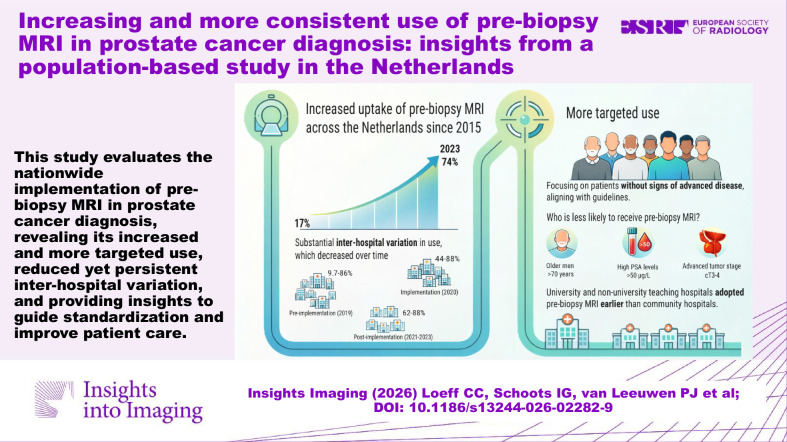

## Introduction

In recent years, MRI has transformed the prostate cancer (PCa) diagnostic pathway in men with elevated prostate-specific antigen (PSA) levels. Clinical trials have shown that the MRI-based diagnostic pathway improves detection of clinically significant PCa, while reducing the detection of clinically insignificant PCa, compared to the conventional diagnostic workup of systematic ultrasound-guided 10- to 12-core biopsies [1–3]. In addition, by limiting biopsies to patients with a positive pre-biopsy MRI (i.e., PI-RADS ≥ 3), the risk of potential side effects can be reduced, as unnecessary biopsies may be avoided in up to 2–49% of men with a clinical suspicion of PCa [1–3]. Consequently, (inter)national guidelines currently recommend performing MRI prior to biopsy decision-making [4, 5]. However, the promising results of the MRI-based pathway are derived from studies involving selected patients, in which interpretation of MRI images was typically conducted by experienced radiologists, and prostate biopsies were performed by experienced urologists. As such, it remains uncertain how results from clinical trials can be translated to daily clinical practice [6, 7]. Therefore, the uptake of pre-biopsy MRI in routine clinical practice should be evaluated, as comprehensive population-level data remain limited [8, 9].

The population-based ADMINISTRATE (Advanced Diagnostic Modalities in Imaging Impacting on diagnosiS, TReatment And paTient outcome) project was initiated to evaluate the use and clinical impact of MRI as a primary staging modality in PCa diagnosis in the Netherlands. As part of this project, the present study provides insight into the uptake of pre-biopsy MRI in the Netherlands between 2015 and 2023, examines patient-, tumor-, and hospital-related factors associated with its use, and assesses inter-hospital variation.

## Patients and methods

All patients diagnosed with histologically confirmed primary PCa in the Netherlands were identified through the Netherlands Cancer Registry (NCR). The main study cohort consisted of patients diagnosed between July 2019 and December 2023. Additionally, a historical cohort comprised patients diagnosed with PCa between October 2015 and April 2016. Patient (e.g., age, comorbidity), tumor (e.g., year of diagnosis, PSA level, disease stage), and hospital characteristics were obtained from the NCR. These data are routinely collected from electronic health records by trained data managers following standardized protocols. If needed, when patients have been referred during their diagnostic (or treatment) trajectory, records from multiple hospitals are consulted. Since July 2019, aligned with guideline updates and as part of the ADMINISTRATE project, this standardized approach has been expanded to include the collection of data on the use of MRI in the diagnostic pathway of PCa. Additionally, MRI data for the historical cohort were collected as part of the ProZIB-study [10]. Comorbidity information was only available for the historical cohort and for a random sample (17%) of the total PCa population diagnosed between 2019 and 2022. Patients were excluded if PCa was incidentally detected in surgical specimens (*n* = 4424), if diagnosed at one of the two specific hospitals where the additional collection of MRI data was not performed (due to contract constraints; *n* = 3544), or if diagnosed at hospitals with fewer than ten PCa diagnoses annually (*n* = 305).

Pre-biopsy MRI was defined as an MRI performed within six months before or on the date of histological confirmation. When patients were referred during their diagnostic trajectory, this definition also included an MRI performed at other institutions. Clinical Tumor-stage (cT-stage) was defined according to the Tumor, Node, and Metastasis classification system of the Union for International Cancer Control [11, 12], and from 2019 onwards was based solely on clinical examination (i.e., digital rectal examination), whereas prior to 2019, imaging was also an accepted method for assessing cT-stage [13]. Hospitals were categorized into community hospitals and university/non-university teaching hospitals (in Dutch: Samenwerkende Topklinische Ziekenhuizen). The Charlson Comorbidity Index (CCI) was determined based on comorbid conditions, without including age adjustments, and classified as follows: 0: no comorbidity, 1–2: mild comorbidity, ≥ 3 moderate/severe comorbidity [14].

The main study cohort was divided into three periods reflecting the implementation of pre-biopsy MRI in the Netherlands: pre-implementation (July–December 2019), implementation (2020), and post-implementation (2021–2023). These periods represent early uptake six months prior to the formal update of national guidelines in January 2020 [5], initial nationwide uptake, and routine clinical use, respectively. The historical cohort, established in the ProZIB-study, served as baseline [10]. During this period, PCa diagnosis primarily relied on systematic biopsies, with MRI not routinely incorporated in the diagnostic pathway.

### Statistical analysis

The uptake of pre-biopsy MRI was calculated as a percentage, with 95% confidence interval (CI) based on the normal approximation of the binomial distribution, for each period and each year in the post-implementation period. Additionally, descriptive analyses were performed to provide insight into the uptake of pre-biopsy MRI by patient, tumor, and hospital characteristics across the periods.

Mixed-effects logistic regression analysis was performed to determine which factors were associated with pre-biopsy MRI use, taking into account the hierarchical structure of patients clustered within hospitals. To assess heterogeneity over time, interaction terms between each factor and the pre-specified periods were included. In this analysis, the implementation period served as a reference. Additionally, a similar exploratory analysis was performed, including only patients for whom comorbidity data were available.

To quantify inter-hospital variation concerning pre-biopsy MRI use in the main cohort, mixed-effects logistic regression models were constructed. Likelihood ratio tests (LRTs) were performed to formally test for inter-hospital variation (i.e., testing for the need of a random intercept). A similar analysis was conducted to evaluate whether inter-hospital variation changed over time, by constructing a single model across all periods and testing for the inclusion of a random slope. All models regarding hospital variation were adjusted for case-mix, i.e., age, PSA level, and cT-stage. From the period-specific models, hospital-specific probabilities, adjusted odds ratios (ORs; with 95% CI), and 75% midranges (i.e., 12.5th–87.5th percentiles) of pre-biopsy MRI use were derived [15].

Two exploratory analyses were performed to further assess inter-hospital variation in the post-implementation period. The first was restricted to patients for whom comorbidity data were available. The second (a univariable analysis), included a more homogeneous group of patients, aged ≤ 80 years, with a PSA ≤ 20 µg/L, and < cT3. These patients are most likely to benefit from curative therapy and, therefore, from MRI assessment.

All analyses were conducted using SAS version 9.4. Approval of an institutional ethics committee for the current study was not required by Dutch law. This study was approved by the Privacy Review Board of the NCR (K23.104).

## Results

**Fig. 1:**
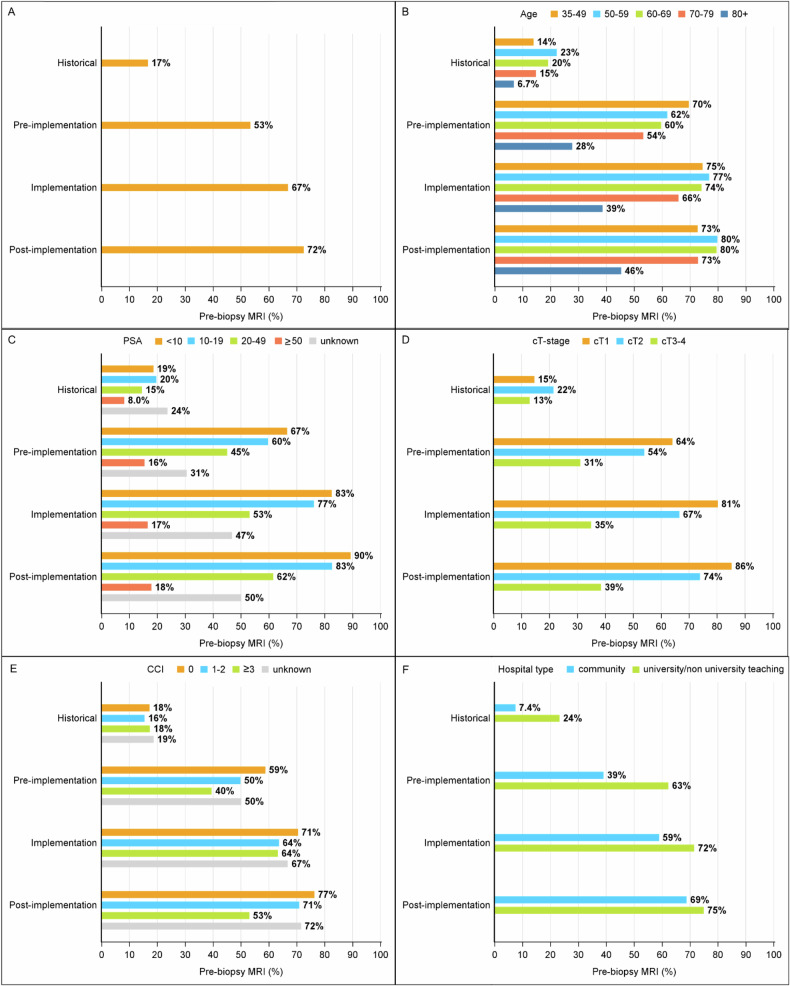
The uptake of pre-biopsy MRI in men with biopsy-proven PCa over time (**A**), by age (**B**), PSA levels (**C**), cT-stage (**D**), CCI (**E**), and hospital type (**F**)

In total, 56,170 men with biopsy-proven PCa were included in this study (main cohort: *n* = 50,987; historical cohort: *n* = 5183). Cohort characteristics by MRI-related period are presented in the Supplementary (S) Table 1. Pre-biopsy MRI use increased from 17% (95% CI: 16–18%) in the historical period to 53% (95% CI: 52–55%) in the pre-implementation period, and 67% (95% CI: 66–68%) in the implementation period. After 2020, further uptake of pre-biopsy MRI was limited, increasing only modestly from 71% (95% CI: 70–71%) in 2021 to 74% (95% CI: 73–74%) in 2023 (Fig. 1a). Figure 1b–f shows the observed pre-biopsy MRI use stratified by patient, tumor, and hospital characteristics across the MRI-related periods, clearly indicating an increase in all subgroups, although the extent of the increase varied. These findings were confirmed by the results from the mixed-effects logistic regression analysis shown in Fig. S1, which presents the estimated pre-biopsy MRI use compared to the reference period by age, PSA level, cT-stage, and hospital type.

### Factors associated with pre-biopsy MRI use

**Fig. 2:**
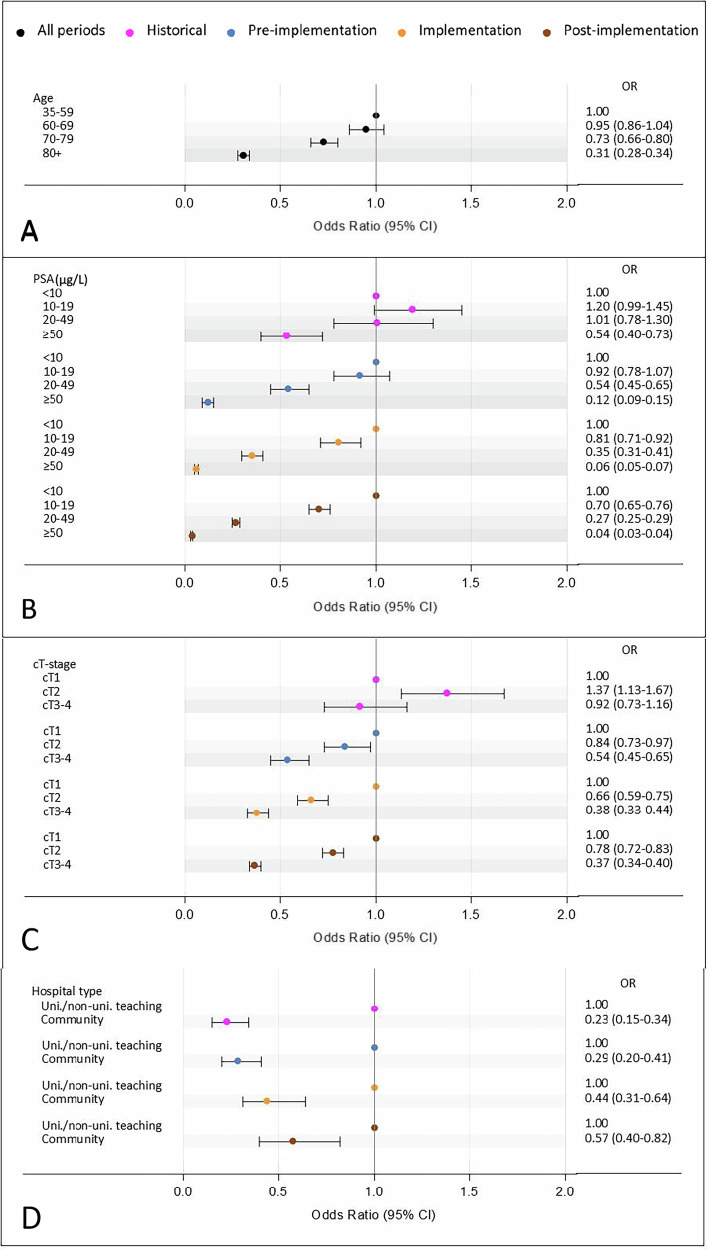
The association between age (**A**), PSA level (**B**), cT-stage (**C**), hospital type (**D**), and pre-biopsy MRI use. Heterogeneity, i.e., statistically significant variation across MRI-related periods, was observed for all factors, except for age, as reflected in the period-specific ORs. These ORs represent the association between each factor and the likelihood of receiving pre-biopsy MRI relative to the reference category. OR = 1 and OR > 1 indicate a higher likelihood of receiving pre-biopsy MRI, while an OR < 1 indicates a lower likelihood

We evaluated whether age, PSA level, cT-stage and hospital type were associated with pre-biopsy MRI use, and whether these associations showed heterogeneity across MRI-related periods (Fig. 2). Men older than 70 years, those with a higher risk of locally advanced disease (i.e., PSA > 50 µg/L or cT3-4 disease), and those diagnosed in community hospitals were less likely to undergo pre-biopsy MRI compared to men younger than 60 years, those with a PSA < 10 µg/L, cT1 disease, and diagnosed in university/non-university teaching hospitals, respectively.

The exploratory analysis, including only patients with comorbidity data (*n* = 10,816), indicated that pre-biopsy MRI was used less frequently in patients with CCI 1 or higher compared to patients without comorbidities (CCI = 0; data not shown).

Heterogeneity across the MRI-related periods was observed for all factors, except for age (Fig. 2). From the pre-implementation period onward, higher PSA levels were consistently associated with a lower probability of receiving a pre-biopsy MRI. In the historical period, however, only patients with a PSA above 50 µg/L were less likely to undergo MRI compared to those with low PSA (< 10 µg/L) (Fig. 2b). Similarly, from the pre-implementation period onward, palpable and advanced disease (i.e., cT2-4) were associated with reduced MRI use compared to non-palpable disease (cT1) (Fig. 2c). Patients diagnosed in community hospitals were less likely to undergo a pre-biopsy MRI compared to those diagnosed in university/non-university teaching hospitals. This difference became less pronounced over time (Fig. 2d).

### Inter-hospital variation in pre-biopsy MRI use

**Fig. 3:**
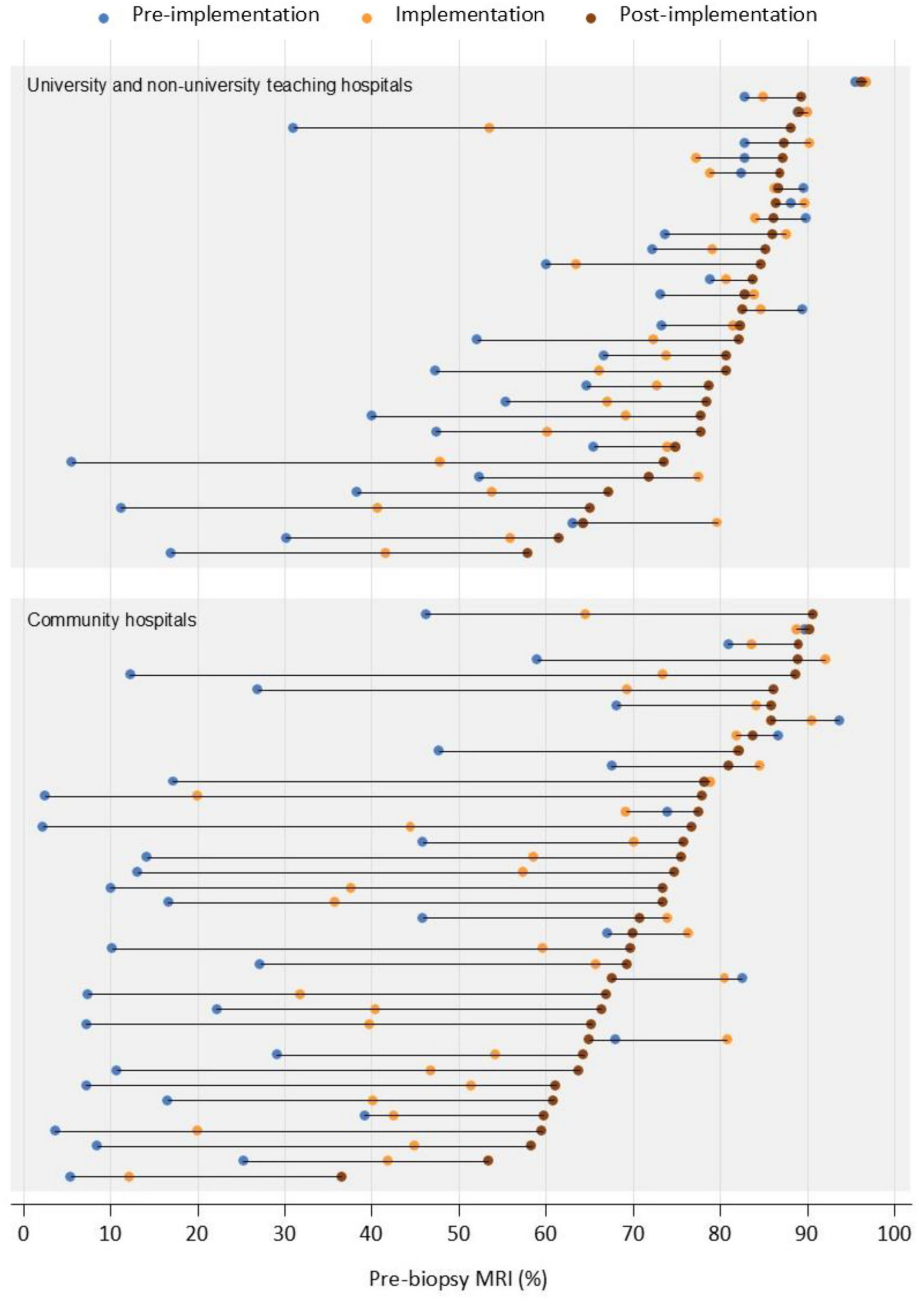
Case-mix (age, PSA, and cT-stage) adjusted probabilities of pre-biopsy MRI use (*x*-axis), analyzed per period, displayed per hospital in the Netherlands (*y*-axis), and grouped by hospital type

**Fig. 4:**
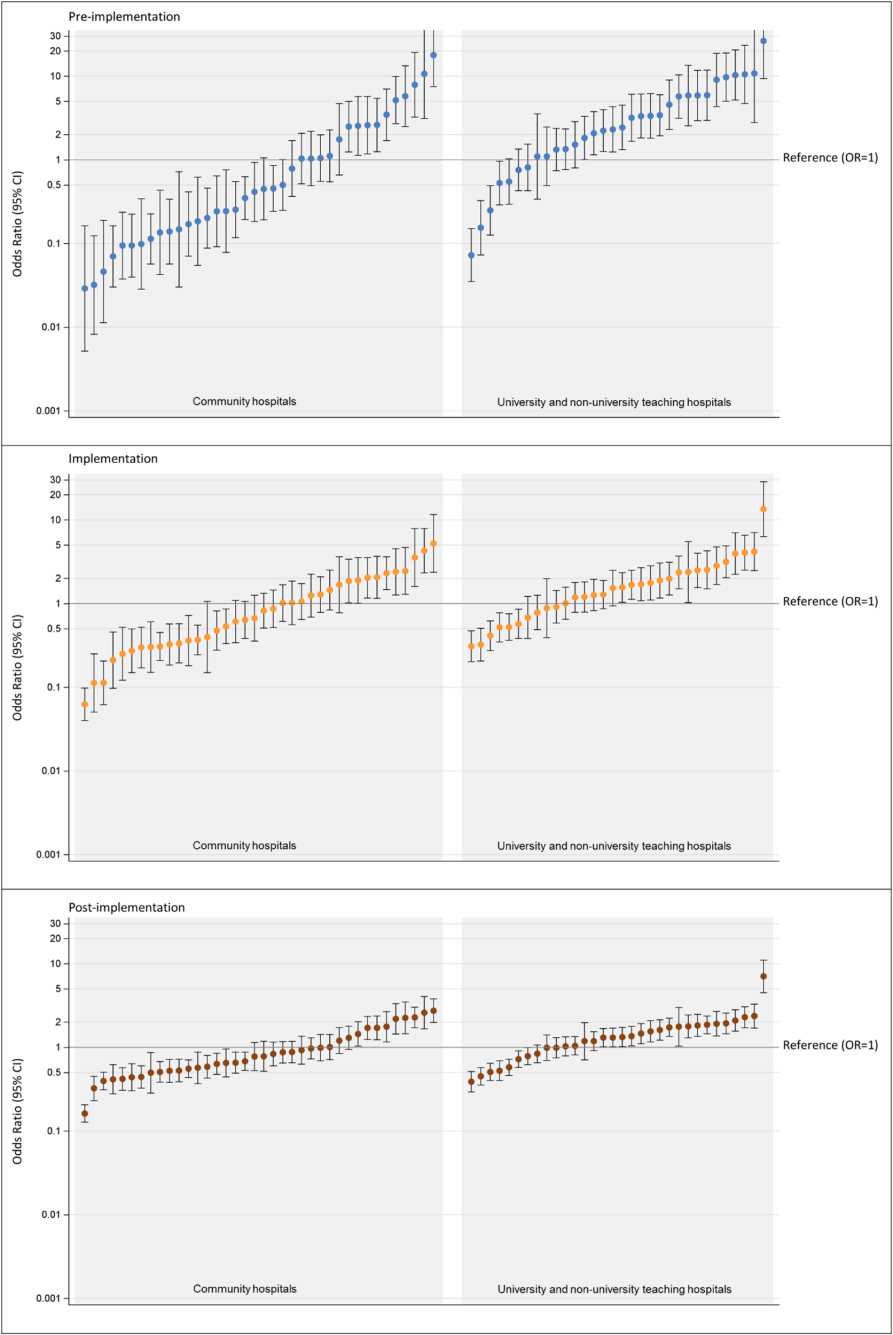
Case-mix (age, PSA, and cT-stage) adjusted ORs with 95% confidence intervals (*y*-axis) for pre-biopsy MRI use, analyzed per period, displayed per hospital (*x*-axis), and grouped by hospital type. Hospitals are displayed in ascending order by period; individual hospital orders may vary

Hospital-specific case-mix adjusted probabilities of pre-biopsy MRI use are presented in Fig. 3. Although substantial inter-hospital variation persisted across all periods (*p* < 0.0001), this variation decreased over time (p < 0.0001). The estimated 75% midranges of pre-biopsy MRI uptake rates were 9.7–86%, 40–88%, and 62–88%, in the pre-implementation, implementation, and post-implementation period, respectively. Figure 4 presents the hospital-specific adjusted ORs of pre-biopsy use for each period, using the nationwide average as reference. Pre-biopsy MRI use was significantly lower than the nationwide average in 39–53% of community hospitals (*n* = 15–20) and 13–22% of university/non-university teaching hospitals (*n* = 4–7). Conversely, significantly higher use was observed in 24–26% of community hospitals (*n* = 9–10) and 50–63% of university/non-university teaching hospitals (*n* = 16–20). The exploratory analysis, including only patients with comorbidity data (*n* = 2838), yielded similar results (data not shown).

The estimated 75% midrange of pre-biopsy MRI uptake rates increased further to 80–94% in patients aged ≤ 80 years, with a PSA ≤ 20 µg/L, and < cT3, i.e., those patients most likely to benefit from MRI (*n* = 23,656, 67% of the total population in the post-implementation period).

## Discussion

This comprehensive, nationwide evaluation of pre-biopsy MRI use in the diagnostic pathway of primary PCa across the Netherlands, revealed a marked increase between 2015 and 2023. Although substantial inter-hospital variation persisted, pre-biopsy MRI use became more consistent across hospitals over time. Pre-biopsy MRI use increased regardless of age, PSA level, cT-stage, and comorbidities. However, older patients, those with a higher PSA or advanced disease stage, and those with comorbidities were consistently less likely to undergo pre-biopsy MRI than younger, healthier patients with more favorable tumor characteristics. Furthermore, from the implementation period onward, patients with a PSA ≥ 10 µg/L or cT2-4-stage were increasingly less likely to receive a pre-biopsy MRI compared to those with a PSA < 10 µg/L and cT1-stage, respectively, reflecting a growing emphasis on risk-stratification.

The growing integration of MRI into the PCa diagnostic pathway has also been observed in other European countries and in the United States (US). In Denmark, for example, 52% of newly diagnosed PCa patients underwent MRI in 2022, representing a substantial increase from 22% in 2020 and 29% in 2021 [16]. A similar upward trend in MRI uptake was observed in Sweden between 2018 and 2020 [17]. In the US, a retrospective single-center study reported an increase from 56% in 2018 to 89% in 2021 among men with a PSA of 2–20 µg/mL [18]. Consistent with our findings, previous studies also showed that men receiving pre-biopsy MRI tend to be younger and have lower PSA levels [17–19].

In men suspected of locally advanced PCa (i.e., PSA > 50 µg/L and/or cT3-4), pre-biopsy MRI for a diagnostic purpose was often omitted, aligning with recent EAU PCa guideline updates [4]. Previous studies have shown that in men with a PSA ≥ 10 µg/L and a suspicious digital rectal examination, pre-biopsy MRI and targeted biopsies offer limited benefit, as MRI is rarely negative and systematic biopsies carry minimal risk of missing clinically significant PCa [20–22]. In these men, metastatic screening is recommended, and alternative imaging modalities such as prostate-specific membrane antigen positron emission tomography/computed tomography (PSMA PET/CT) are increasingly used [4, 23–25].

Next to the increasing emphasis on risk-stratification, we observed that university/non-university teaching hospitals adopted the pre-biopsy MRI earlier than community hospitals. Although this gap narrowed over time, it remained present, likely due to the greater involvement of university/non-university teaching hospitals in multicenter studies evaluating the MRI-based diagnostic pathway, combined with a specialized staff, improved MRI infrastructure, and MRI availability [26, 27].

Beyond differences in adoption rates, substantial inter-hospital variation in pre-biopsy MRI use persisted after adjusting for age, PSA, cT-stage, and comorbidities. This might suggest that other unmeasured patient- or tumor-related factors continue to influence pre-biopsy MRI use. However, physician preferences, regional or hospital-specific guidelines, and differences in the organization of care pathways, such as the presence of rapid diagnostic centers that enable same-day workups and streamline diagnostic processes, may also contribute to this variation [28].

Pre-biopsy MRI use was highest among patients aged ≤ 80 years, with a PSA ≤ 20 µg/L, and < cT3, i.e., those most likely to benefit from curative therapy (75% midrange of 80–94% vs 62–88% in the total PCa population). However, a substantial proportion of patients fulfilling these criteria did not receive a pre-biopsy MRI. This highlights the need for discussion, nationwide and within PCa and/or oncology networks, to further optimize the diagnostic pathway of primary PCa.

The increased MRI use in PCa diagnosis, as well as in future screening programs, combined with an aging population and subsequent higher PCa incidence, presents a substantial future challenge for the health care system. While advancements such as artificial intelligence and the use of biparametric MRI instead of multiparametric MRI could help optimize MRI workflows and improve efficiency [29–31], growing personnel shortages and potential scanner scarcity remain. To meet the increasing demand for MRI in the future, a more targeted, risk-stratified approach appears to be essential [32].

Some limitations should be noted. It was a deliberate methodological choice to embed the ADMINISTRATE project, of which this study is a part, in the NCR. While the NCR is well-suited for evaluating trends over time among PCa patients, it does not capture data on men with suspected PCa who did not receive a PCa diagnosis. As a result, we could not assess the proportion of men who underwent pre-biopsy MRI without subsequent confirmation of PCa, nor evaluate changes in the rate of potentially unnecessary biopsies, which would provide data on the real-world effectiveness of the MRI-based pathway. Nonetheless, a study performed in the Netherlands, using data from the nationwide pathology registry, showed that in the period between 2015 and 2020, the proportion of non-malignant biopsies decreased from approximately 50% to 30% [33]. Unfortunately, the NCR lacks information on general health status, and life expectancy, which may influence pre-biopsy MRI use [34]. As a result, the case-mix adjustment in the analyses evaluating inter-hospital variation may be incomplete.

This population-based study demonstrated the substantial increase in pre-biopsy MRI use across the Netherlands since 2015 among patients with biopsy-confirmed PCa. Over time, inter-hospital variation declined, and MRI use became more targeted, focusing on patients without signs of advanced disease. Nonetheless, significant differences between hospitals remained, and a considerable proportion of eligible patients did not receive a pre-biopsy MRI. Enhancing clinician awareness, addressing structural barriers, ensuring equitable access across hospitals, and adopting a risk-stratified approach are essential for optimizing diagnostic pathways and improving outcomes for men with suspected PCa.

## Supplementary information

ELECTRONIC SUPPLEMENTARY MATERIAL

## Data Availability

All data used for this study can be requested from the NCR. All data requests are reviewed by the supervisory committee of the NCR for compliance with the NCR objectives and (inter)national (privacy) regulation and legislation (https://iknl.nl/en/ncr/apply-for-data).

## Abbreviations

CCI: Charlson comorbidity index
cT-stage: Clinical tumor-stage
NCR: Netherlands Cancer Registry
OR: Odds ratio
PCa: Prostate cancer
PSA: Prostate-specific antigen
