# Supplementary material for: Increasing and more consistent use of pre-biopsy MRI in prostate cancer diagnosis: insights from a population-based study in the Netherlands
Source: Insights Imaging. 2026 May 28;17:143. doi: 10.1186/s13244-026-02282-9 (PMC13219667; doi:10.1186/s13244-026-02282-9)
Supplement: Supplementary file 1 — ELECTRONIC SUPPLEMENTARY MATERIAL [file 13244_2026_2282_MOESM1_ESM.pdf]

Increasing and more consistent use of pre-biopsy MRi in prostate cancer diagnosis: insights from a population-based study in the Netherlands.

ELECTRONIC SUPPLEMENTARY MATERIAL

Supplementary table 1. Cohort characteristics by period.

	Historical (n = 5,183)	Pre-implementation (n = 5,530)	Implementation (n = 10,130)	Post-implementation (n = 35,327)
**Pre-biopsy MRI**, n (%)				
No	4,327 (83%)	2,586 (47%)	3,368 (33%)	9,764 (28%)
Yes	856 (17%)	2,944 (53%)	6,762 (67%)	25,563 (72%)
Interval (days) between MRI and histological confirmation, median (IQR)*	15 (1-29)	20 (10-37)	20 (11-35)	22 (13-35)
**Age**, n (%)				
Mean (SD)	69.2 (7.6)	70.4 (7.6)	70.3 (7.5)	70.8 (7.5)
35-49	29 (0.6%)	36 (0.7%)	39 (0.4%)	135 (0.4%)
50-59	503 (9.7%)	436 (7.9%)	855 (8.4%)	2,628 (7.4%)
60-69	2,194 (42%)	1,876 (34%)	3,457 (34%)	11,663 (33%)
70-79	2,010 (39%)	2,570 (46%)	4,746 (47%)	16,950 (48%)
80+	447 (8.6%)	612 (11%)	1,033 (10%)	3,951 (11%)
**PSA level**, n (%)				
Median (IQR)	10.5 (6.8-25.2)	10.4 (6.6-26.3)	10.2 (6.7-24.0)	10.2 (6.6-24.9)
<10	2,433 (47%)	2,628 (48%)	4,880 (48%)	16,875 (48%)
10-19	1,195 (23%)	1,193 (22%)	2,320 (23%)	7,969 (23%)
20-49	717 (14%)	752 (14%)	1,316 (13%)	4,624 (13%)
≥50	821 (16%)	934 (17%)	1,569 (15%)	5,699 (16%)
Unknown	17 (0.3%)	23 (0.4%)	45 (0.4%)	160 (0.5%)
**cT-stage**, n (%)				
cT1	1,583 (31%)	2,532 (46%)	4,915 (49%)	17,257 (49%)
cT2	1,958 (38%)	1,741 (31%)	3,187 (31%)	11,143 (32%)
cT3-4	1,642 (32%)	1,257 (23%)	2,028 (20%)	6,927 (20%)
**Charlson Comorbidity Index**, n (%)				
0	3,006 (58%)	585 (59%)	1,086 (60%)	1,643 (58%)
1-2	1,459 (28%)	258 (26%)	491 (27%)	825 (29%)
≥3	578 (11%)	139 (14%)	231 (13%)	363 (13%)
Unknown	140 (2.7%)	2 (0.2%)	3 (0.2%)	7 (0.2%)
Not collected**, n	-	4,546	8,319	32,489
**Hospital type**, n (%)				
Community	2,173 (42%)	2,112 (38%)	3,737 (37%)	13,942 (39%)
University and non-university teaching	3,010 (58%)	3,418 (62%)	6,393 (63%)	21,385 (61%)

**The date of MRI was missing in 247 (<1%) cases; in all of these cases there was a record of MRI-guided biopsies. **Comorbidity data were only available for the historical cohort and for a random sample of the total PCa population diagnosed between 2019-2022 (n=10,816).*

Supplementary figure 1. Forest plot illustrating the temporal increase of pre-biopsy MRI use across all age groups (A), PSA levels (B), cT-stages (C), and hospital types (D).

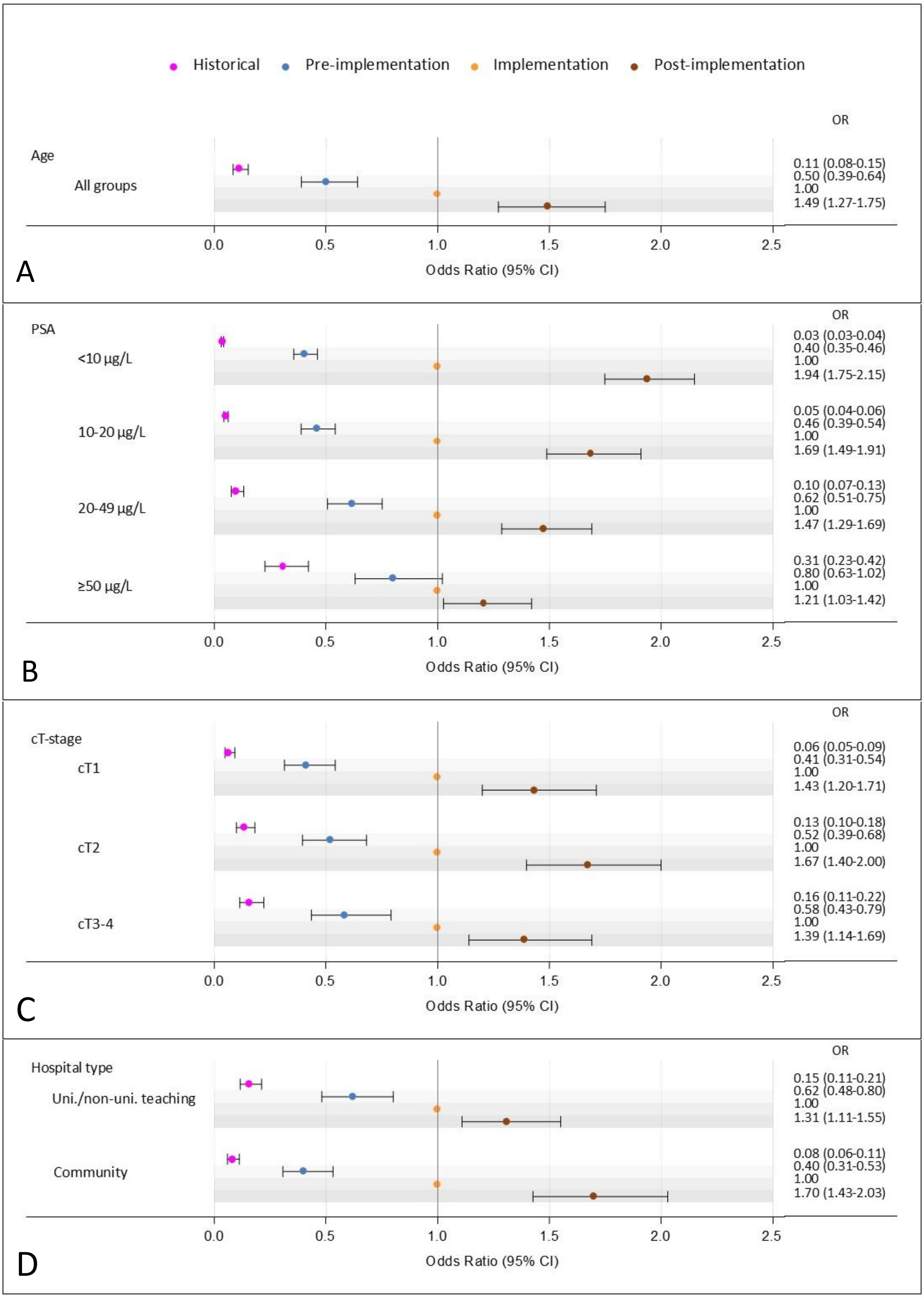

OR = Odds Ratio. MRI = magnetic resonance imaging. PSA = prostate-specific antigen. cT-stage = clinical-Tumor stage. Uni./non-uni. teaching = University/non-university teaching. The ORs for the unknown PSA category are not presented.
